# Dual-Energy CT in Head and Neck Imaging

**DOI:** 10.1007/s40134-017-0213-0

**Published:** 2017-03-29

**Authors:** Elise D. Roele, Veronique C. M. L. Timmer, Lauretta A. A. Vaassen, Anna M. J. L. van Kroonenburgh, A. A. Postma

**Affiliations:** 1 Department of Radiology, Maastricht University Medical Centre+, PO Box 5800, 6202 AZ Maastricht, The Netherlands; 2 Department of Cranio and Maxillofacial Surgery, Maastricht University Medical Centre+, PO Box 5800, 6202 AZ Maastricht, The Netherlands

**Keywords:** Dual-energy CT, Spectral CT, Head and neck cancer, Lymph node imaging, Parathyroid adenoma, Metal artifact reduction

## Abstract

**Purpose of Review:**

To explain the technique of Dual-energy CT (DECT) and highlight its applications and advantages in head and neck radiology.

**Recent Findings:**

Using DECT, additional datasets can be created next to conventional images. In head and neck radiology, three material decomposition algorithms can be used for improved lesion detection and delineation of the tumor. Iodine concentration measurements can aid in differentiating malignant from nonmalignant lymph nodes and benign posttreatment changes from tumor recurrence. Virtual non-calcium images can be used for detection of bone marrow edema. Virtual mono-energetic imaging can be useful for improved iodine conspicuity at lower keV and for reduction of metallic artifacts and increase in signal-to-noise ratio at higher keV.

**Summary:**

DECT and its additional reconstructions can play an important role in head and neck cancer patients, from initial diagnosis and staging, to therapy planning, evaluation of treatment response and follow-up. Moreover, it can be helpful in imaging of infections and inflammation and parathyroid imaging as supplementary reconstructions can be obtained at lower or equal radiation dose compared with conventional single energy scanning.

## Introduction

Imaging is a cornerstone of the diagnostic work-up of patients with suspected head and neck pathology, especially in the diagnosis and staging of head and neck cancer, but also in inflammatory processes, abscesses, and lymph node imaging. The available imaging arsenal expanded in the recent decades from conventional radiography, fluoroscopy, and ultrasound to more sophisticated imaging modalities like CT and MRI including advanced CT and MR techniques as well as hybrid imaging as are PET/CT and PET/MR.

Starting in the late eighties, CT shifted from sequential scanning to spiral or helical scanning. This was soon followed by the introduction of multidetector row imaging. At the moment CT scanners with detectors up to 320 rows are clinically available. These developments increased the imaging quality as regard to temporal and spatial resolution. The ongoing development in the computational abilities, e.g. in iterative reconstructions, increases the quality and clinical applications of CT even further.

It has only been since the last decade that Dual-energy CT (DECT) became clinically available. By means of two X-ray spectra instead of one, DECT offers increased capabilities and advantages over single energy scanning methods, such as the potential for material characterization and differentiation and the calculation of virtual mono-energetic reconstructions. In the early years applications of CT were mainly in thoracic, abdominal and vascular imaging fields, for example, automated bone removal in CTA, quantification of the amount of iodine in renal masses, and detection of perfusion defects in CTA in patients with pulmonary embolism [1–6]. Later, this was followed by a more widespread implementation in other areas of the body as in the imaging of gout [7, 8]. Initially brain and head and neck applications fell somewhat behind, both now, however, trying to catch up with others [9–11].

In this article, we will explain the basics of the technique of Dual-energy CT, and its applications and advantages in head and neck radiology.

## DECT Technique

**Fig. 1:**
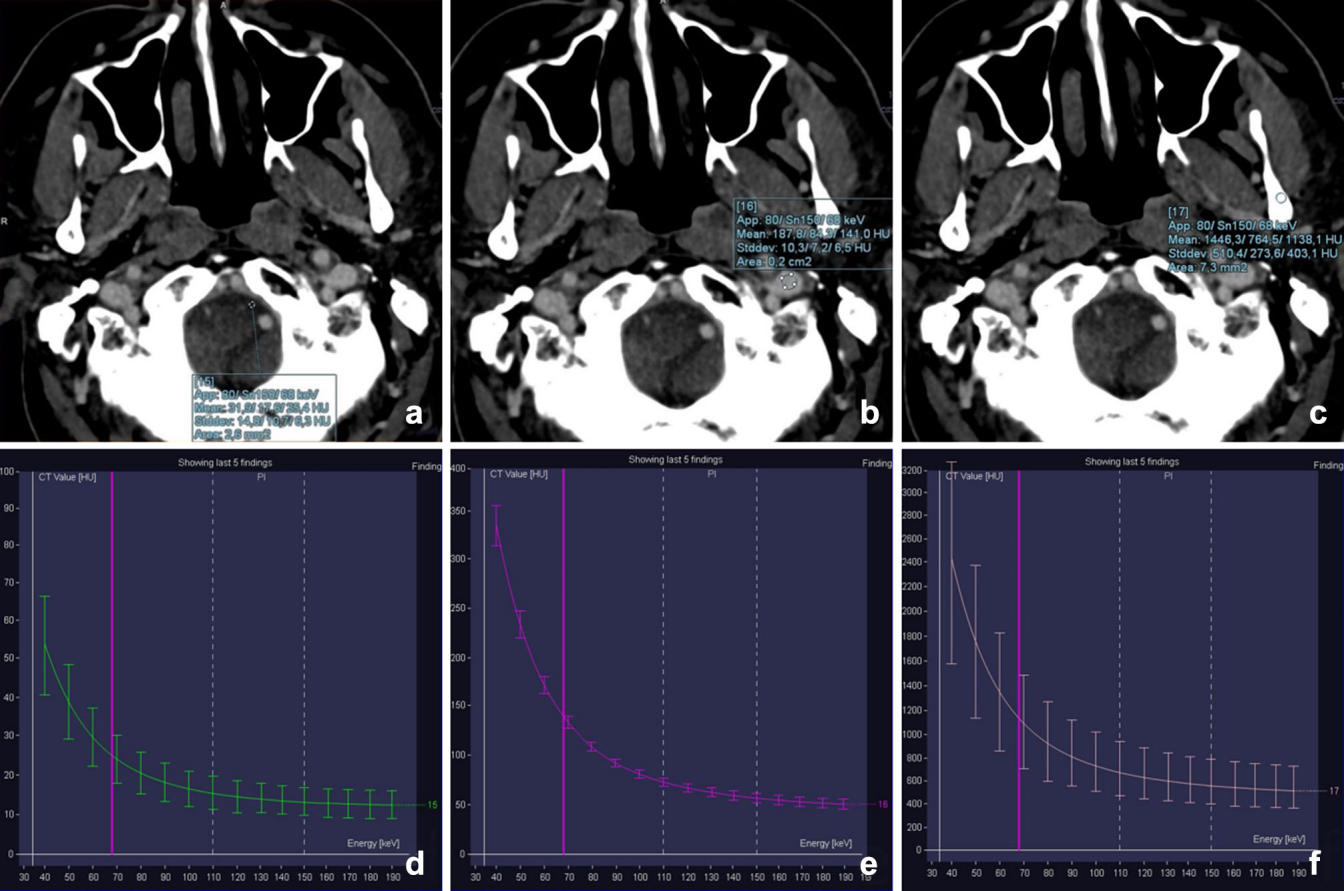
The spectral curves depicted from a DECT of the neck are shown for cerebrospinal fluid (water **a**, **d**), vessels (iodine **b**, **e**), and bone (calcium **c**, **f**). Hounsfield units (HU) are plotted against the mono-energetic energies ranging from 30 to 190 keV. Note that the HU scale differs for each plot. At lower energy, the HU increases, especially in calcium and iodine

The density of the tissues in CT is calculated by the attenuation coefficient expressed in Hounsfield units (HU). The difference between the numbers of the photons emitted and that of the detected ones equals the attenuation and is determined by the interaction of photons with and within the tissue. In radiology, these interactions are mainly determined by photon absorption (photoelectric effect) and scattering (Compton effect). The variation in Compton effects at the energy levels used in CT is relatively small across different materials, while the photoelectric effect varies considerably and is strongly dependent on the atomic number (*Z*) of the material and the photon energy ((*Z*/*E*)^3^) (Fig. 1).

**Fig. 2:**
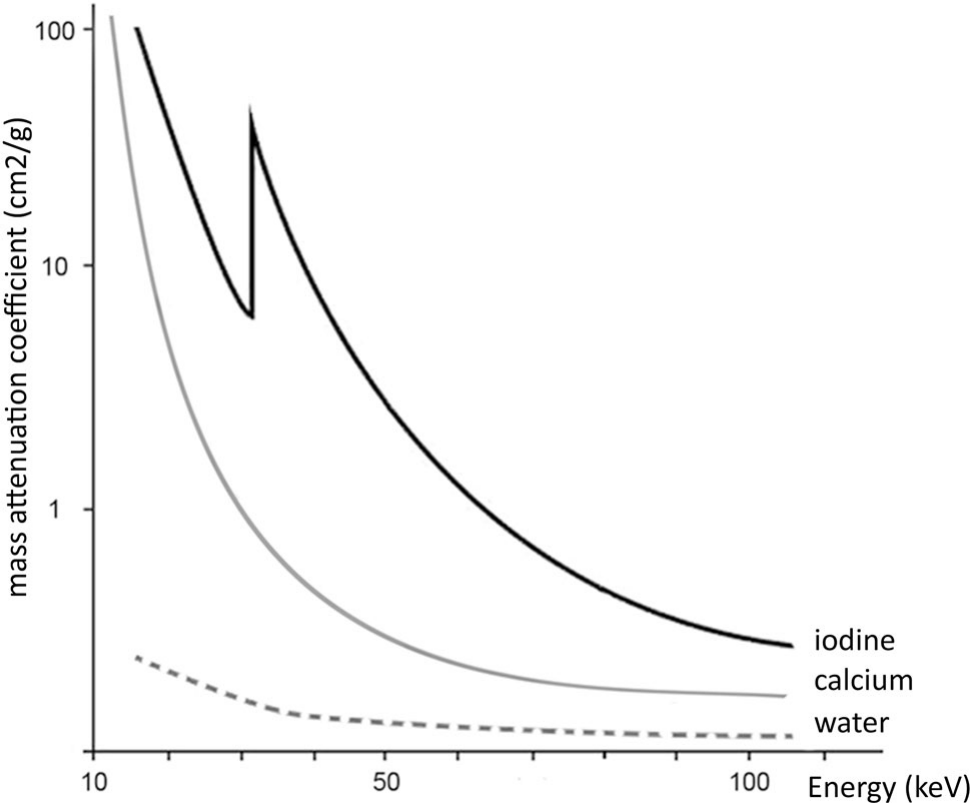
Attenuation curves of iodine, calcium, and water plotted against energy (keV). At lower energy, the attenuation of iodine (*Z* = 53) is increasing with an additional increase at the k-edge. The attenuations of calcium (*Z* = 20) and water are significantly lower than that for iodine, providing the possibility for material differentiation in DECT

The photoelectric effect increases with the increasing atomic number. Commonly used contrast media like iodine (*Z* = 53) and barium (*Z* = 56) have strong photoelectric effects resulting in a high attenuation, especially at the lower energies, because of reaching the k-edge. Most of the tissues in the human body consist of lower effective atomic numbers, such as fat and water, which show relatively weak photoelectric effect and attenuation. In these tissues, the Compton effect prevails. An exception is calcium (*Z* = 20) which has a relative high atomic number compared with the other (soft) tissues of the human body and therefore shows in comparison a higher photoelectric effect and attenuation (Fig. 2).

The attenuation also depends on the photon energy (KeV) and is determined by the maximum voltage level of the X-ray tube (kVp). In Fig. 2, we can visualize that when scanning with two different energies, there is a difference in the attenuation coefficients of the tissues. This difference in attenuation at different energies rests at the basis of DECT scanning.

**Fig. 3:**
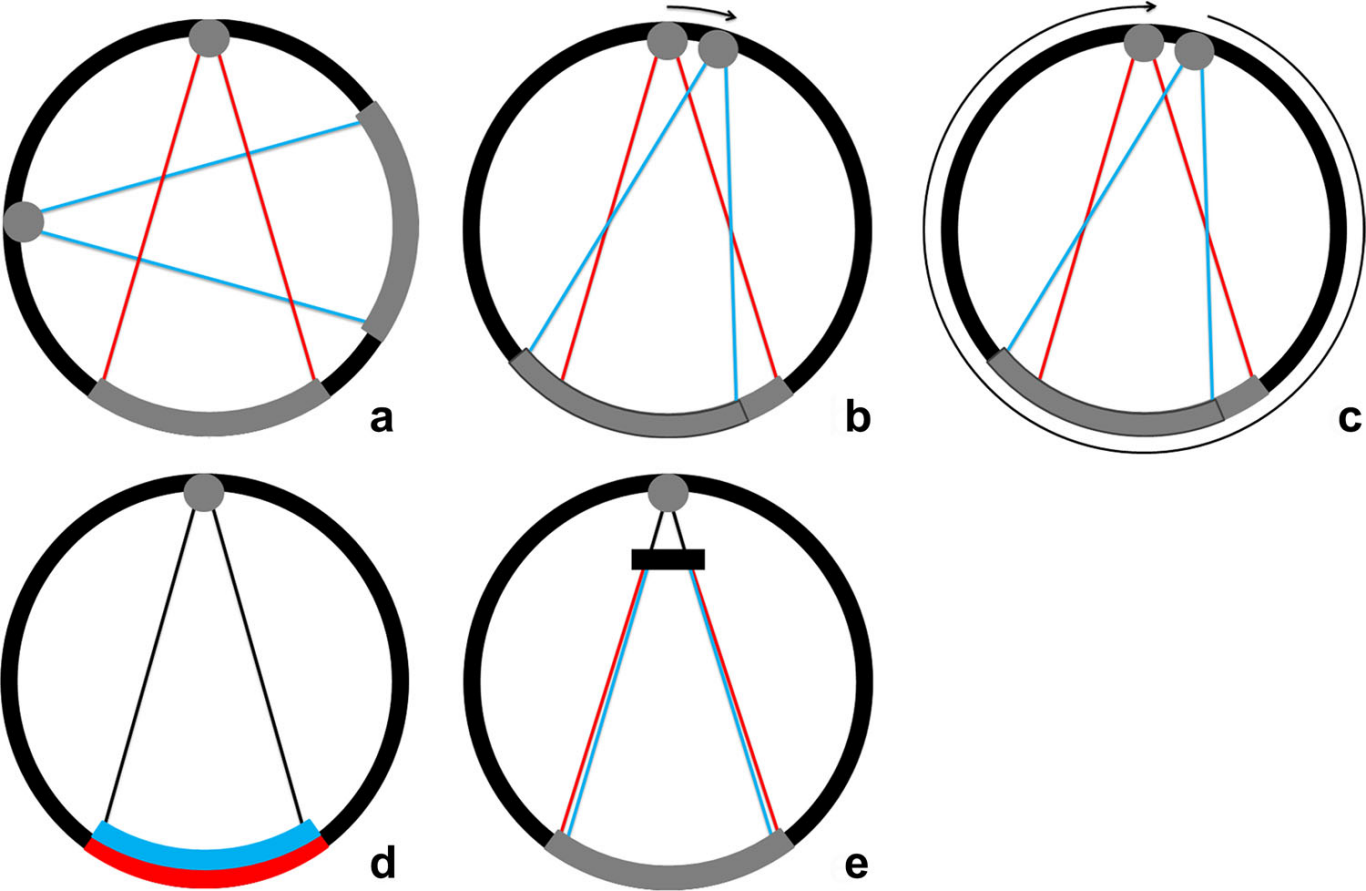
DECT systems. Dual-source dual-energy system (**a**): two separate X-ray tubes and detectors are orthogonally mounted for simultaneous data acquisition and processing. Each tube can be set at different voltage levels. The kVp settings can be adjusted between 70 and Sn150 kVp for the latest generation dual-source scanner (Siemens). Single-source dual-energy system: switching the kVp setting can generate two spectra. One method is fast kV switching (**b**), in which one X-ray tube rapidly switches between low and high kVps (GE). The second method is kVp switching between single rotations (**c**), the so-called dual spiral approach (Toshiba, Siemens). A single detector then processes the information from both voltage levels. Dual-layer system (**d**): a polychromatic spectrum from one tube passes on to a dual-layer detector. The upper layer is sensitive to the low-energy photons, while the second layer processes the high-energy photons. The combination of both detectors creates the combined image (Phillips). Single-source twin-beam system (**e**): the single X-ray beam is pre-filtered between the tube and the patient by gold (Au) and tin (Sn) filter. The 120-kVp X-ray beam is split into high- (Sn) and low-energy (Au) spectra (Siemens)

In single energy CT (SECT) scanners, one single polychromatic energy spectrum is used for imaging, whereas in DECT, two X-ray spectra are needed. In the initial description of DECT by Hounsfield, objects were scanned twice. Today, there are multiple ways to perform DECT scanning [12, 13]. Different DECT techniques include systems with a double X-ray tube, systems with a single tube which can change the kVp setting, with a filter or a dual-layer detector (Fig. 3).

The evaluation of the acquired data by DECT scanning gets reconstructed into low- and high-energy datasets, in which 80 and 140 kVp constitute the frequently encountered combination. The two datasets can be combined to a single mixed-image dataset, also called linear blending or weighted average, resembling a SECT. With a ratio of 0.3 (30% 80 kVp and 70% 140 kVp) a conventional, single energy data image set at 120-kVp acquisition is simulated [3, 14]. By shifting the percentage of contribution of each dataset (linear blending), one can either choose to move to higher-energy contribution for the increased signal-to-noise ratio (SNR) and artifact reduction or a lower-energy contribution for the improved contrast-to-noise ratio (CNR) and lesion conspicuity [12]. Next to linear blending, nonlinear blending functions have been developed to optimize the blending processing [15]. Datasets are mixed via a computational function e.g. a sigmoidal function to maximize contrast and lower noise, which should provide an optimal contrast image [16].

Virtual monochromatic imaging (VMI) allows for image reconstruction at different virtual monochromatic energies instead of using a polychromatic spectrum. At higher virtual energies, beam hardening artifacts can be reduced and SNR is increased. At lower virtual monochromatic energies there is increased conspicuity of iodine (at the cost of lower SNR). These VMI images are reconstructed for a specific purpose and are commonly used in addition to the standard (blended) reconstructions [17–19]. By use of VMI at different energies, a spectral attenuation curve as a function of energy can be plotted [19] (Fig. 1). More recently, advanced algorithms for VMI are allowing an increased SNR at lower virtual energies by using an advanced calculation with the use of a split frequency filter [20, 21].

One of the major strengths of DECT is material differentiation and characterization. Material specific images for identifying or differentiating certain materials/tissues are calculated by using the unique linear attenuation coefficient of the specific materials (fat, calcium, iodine and water). In a 3-material algorithm, the concentration of e.g. iodine or calcium can be calculated. When the iodine concentration is known, it can be subtracted from the mixed dataset to generate a virtual noncontrast (VNC) image. The iodine map can be superimposed in color on the gray-scale image to create fusion images. Similar techniques are used to generate virtual non calcium (VNCa) images.

Next to VMI and material differentiation, the effective Z number and electron density can be calculated from the datasets.

Initially, there have been concerns about higher radiation dose of DECT compared to conventional CT scanners, reported up to three times as high [22]. With advances in technology the radiation dose has lowered and is now comparable to, or even less than, conventional SECT scanners. Either by direct lowering of the dose or the potential of replacing the nonenhanced scan by VNC images and therefore indirect dose reduction [10, 23, 24]. This opens up the wider use of DECT in clinical applications.

## Applications

### Metal Artifact Reduction

Metallic hardware can negatively affect image quality of surrounding tissues because of artifacts. This limitation is especially relevant in imaging of the oral cavity, with the frequent appearance of dental restorations, prosthetics, braces and metallic implants.

**Fig. 4:**
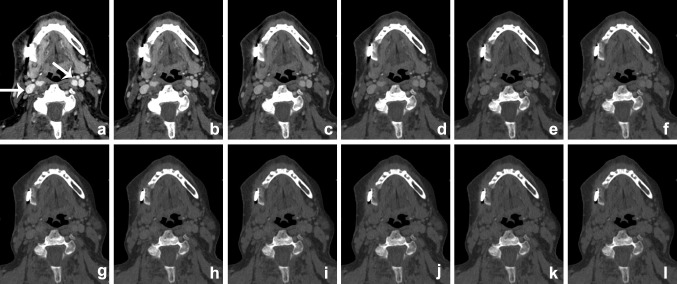
VMI at the range of 40–150 keV (**a**–**l**, 10-keV interval) scanned after administration of iodinated contrast. The patient presented with an osteomyelitis of the mandible and cutaneous fistula (see also Fig. 12). Iodine conspicuity was increased at lower keV, as can be noticed by the increased CNR around the vessels (*arrow*). Higher-energy VMI results in an increase in signal-to-noise (SNR) and a decrease in streak artifacts caused by metallic hardware

**Fig. 5:**
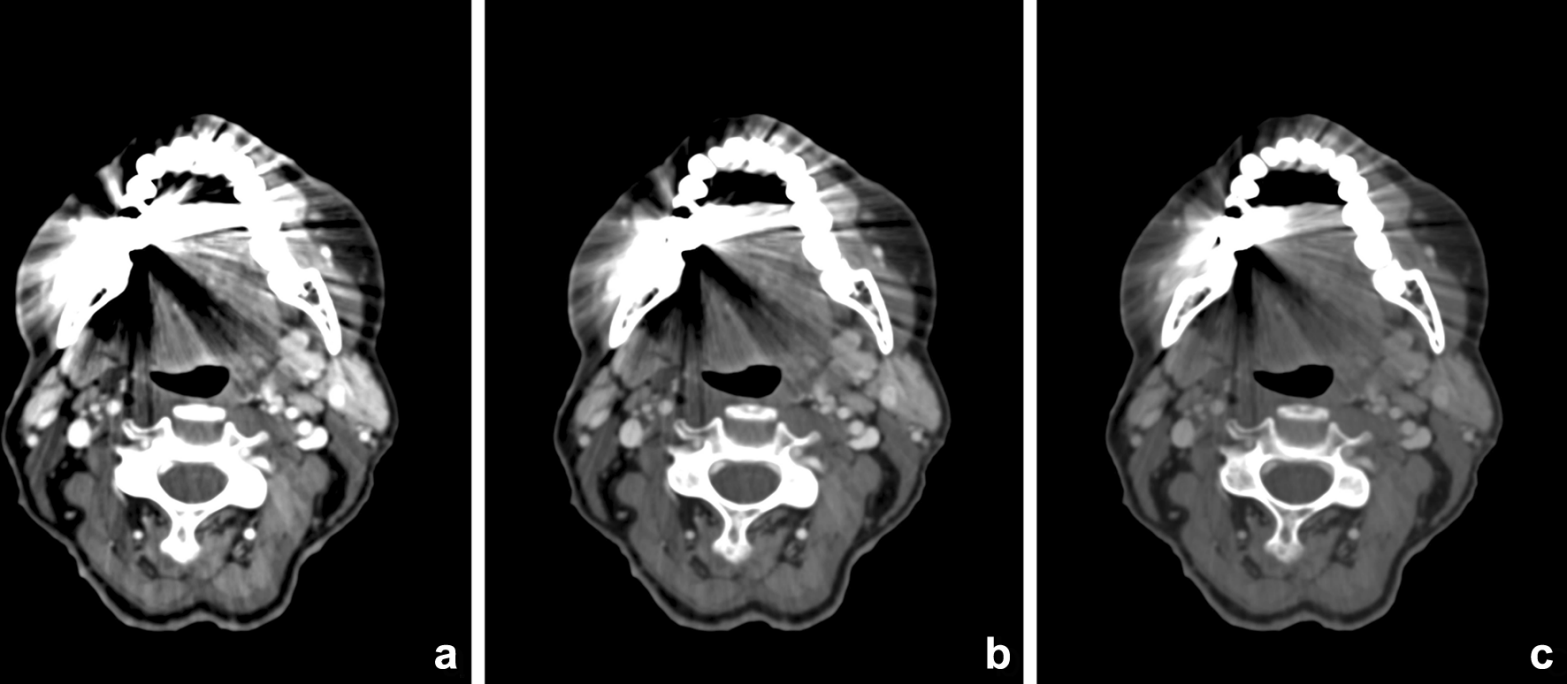
Contrast-enhanced DECT of the oral cavity with VMIs at 55 keV (**a**), 70 keV (**b**), and 100 keV (**c**). Significant beam-hardening artifacts in the oral cavity are present, due to dental fillings. These artifacts are reduced at higher VMI. This is accompanied by an increase of SNR, whereas iodine conspicuity decreases

**Fig. 6:**
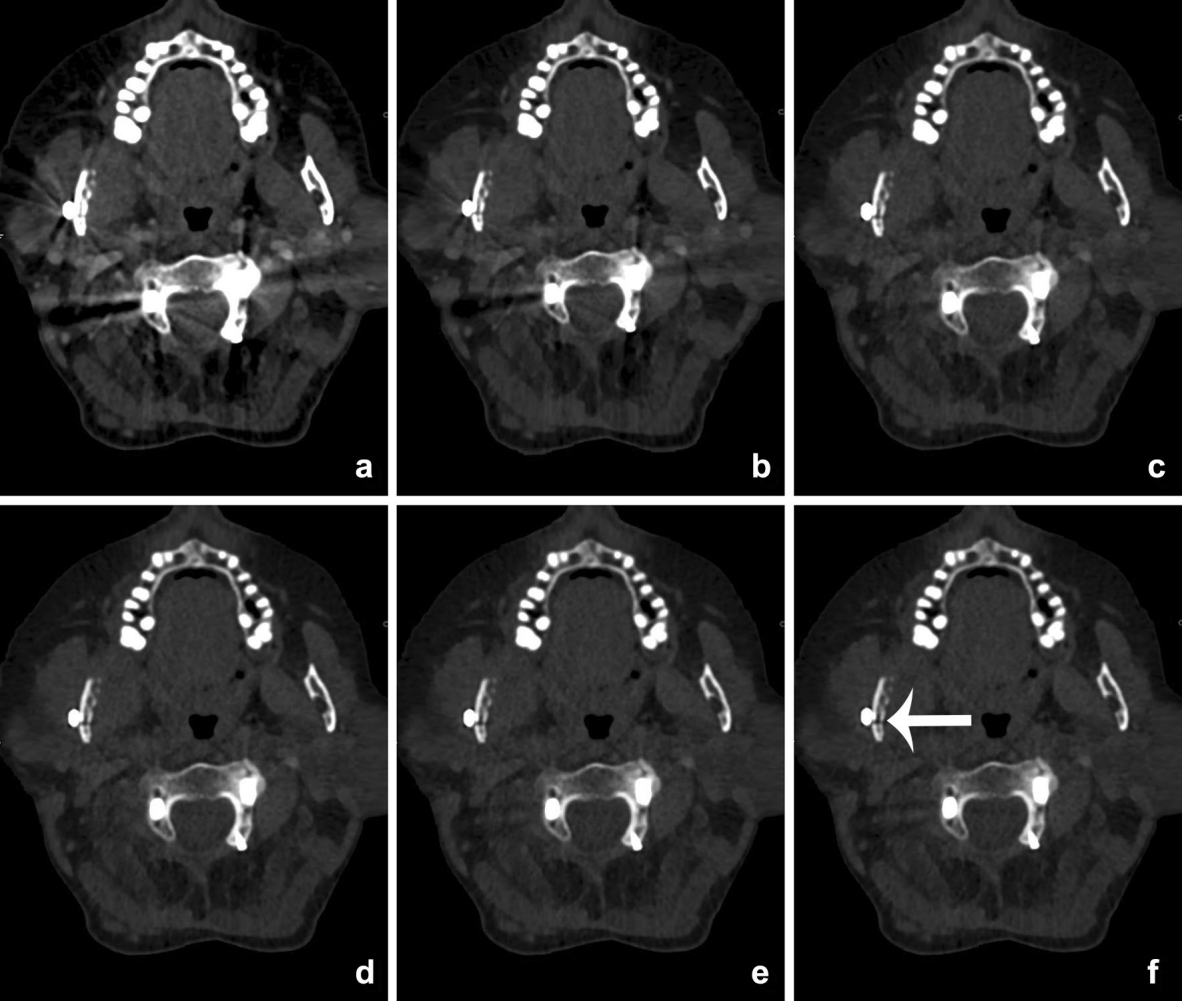
VMIs at 60 (**a**), 80 (**b**), 100 (**c**), 120 (**d**), 140 (**e**), and 160 keV (**f**) of a patient with a cervical spondylodesis and metallic hardware of the mandible (see also Fig. 12). With the increasing virtual mono-energetic energy, further reduction of metallic artifacts of both the cervical spondylodesis and the metallic hardware of the mandible can be seen. At lower keV, streak artifacts obscure pathology at the right mandibular ramus. At higher keV, a fracture becomes visible at the mandible, which was undetectable at the mixed-imaging and low-keV images (*arrow*)

Metal artifacts on CT imaging are mainly caused by photon starvation or beam hardening effects by the high attenuation of metal. Photon starvation occurs when an X-ray beam is completely absorbed by an object and an insufficient number of photons reach the detector to reconstruct an image (zero admission). Beam hardening artifacts are caused by absorption of the low energy photons of the polychromatic X-ray beam. The detected X-ray beam is ‘hardened’, containing higher average energy photons than expected. This results in streaks or dark-like bands around the object [25]. Since the clinical introduction of DECT, this technique has proven to be beneficial in the metal artifact reduction arsenal. Especially reconstructed monochromatic imaging is effective in reducing beam hardening artifacts by the absence of spectral shifts, which are present in polychromatic imaging [26] (Figure 4). VMI reconstructions of high energy levels can reduce metal artifacts and increase image quality [27–34] (Figs. 5, 6). Stolzmann et al. described the use of DECT versus SECT for metal artifact reduction in dental restorations. They found that the use of increasing energy VMI significantly reduced the amount of beam hardening artifacts caused by dental restorations. Image artifacts were lower on VMI than on conventional SECT [34]. Tanaka et al. evaluated different VMIs at 100, 190 keV and fused DECT images, resembling conventional 120-keV CT imaging in living patients with dental implants. They concluded that 100 keV VMI was superior to 190-keV VMI and fused DECT imaging in reducing dark band-like metal artifacts caused by dental implants and additionally resulted in better adjacent bone diagnosis around the implants [27]. In a human cadaver study, De Crop et al. compared metal artifact reduction methods to conventional SECT of the oral cavity. High energy VMI (140 keV) of DECT not only resulted in significant artifact reduction and better image quality but also reduced the low contrast resolution [35]. However, in their study, model based iterative reconstructions (IMAR) seemed to be the most promising metal artifact reduction technique for increasing image quality without adversely affecting contrast resolution [35]. Bongers et al. compared DECT based and iterative metal artifact reduction on hip prosthesis and dental hardware. Although IMAR showed a significantly higher reduction of metal artifacts, compared to VMI of 130-keV DECT images, the combination of DECT and IMAR resulted in a highly significant reduction of metal artifacts compared with IMAR alone [36].

The use of DECT in metal artifact reduction of cervical spinal implants has been evaluated by Guggenberger et al. and Zhou et al. [30, 32]. Both authors concluded that, compared to average weighted 120-kVp image, higher-keV VMI improved image quality and reduced metal artifacts in patients with metal orthopaedic implants. Zhou et al. found the optimal VMI at 130 keV. Furthermore, Guggenberger et al. calculated the individually adjusted mono-energy for optimized image quality (OPTkeV) for different spinal levels and vendors of the spinal implants, which showed a range between 123 and 141 keV [32].

The optimal energy levels for metal artifact reduction are generally found between 100 and 140 keV [28–32, 35–37] (Fig. 6). However, the extent of artifact reduction also depends on location, geometry and material composition of the implant.

### Head and Neck Oncology

#### Primary Tumor Delineation

**Fig. 7:**
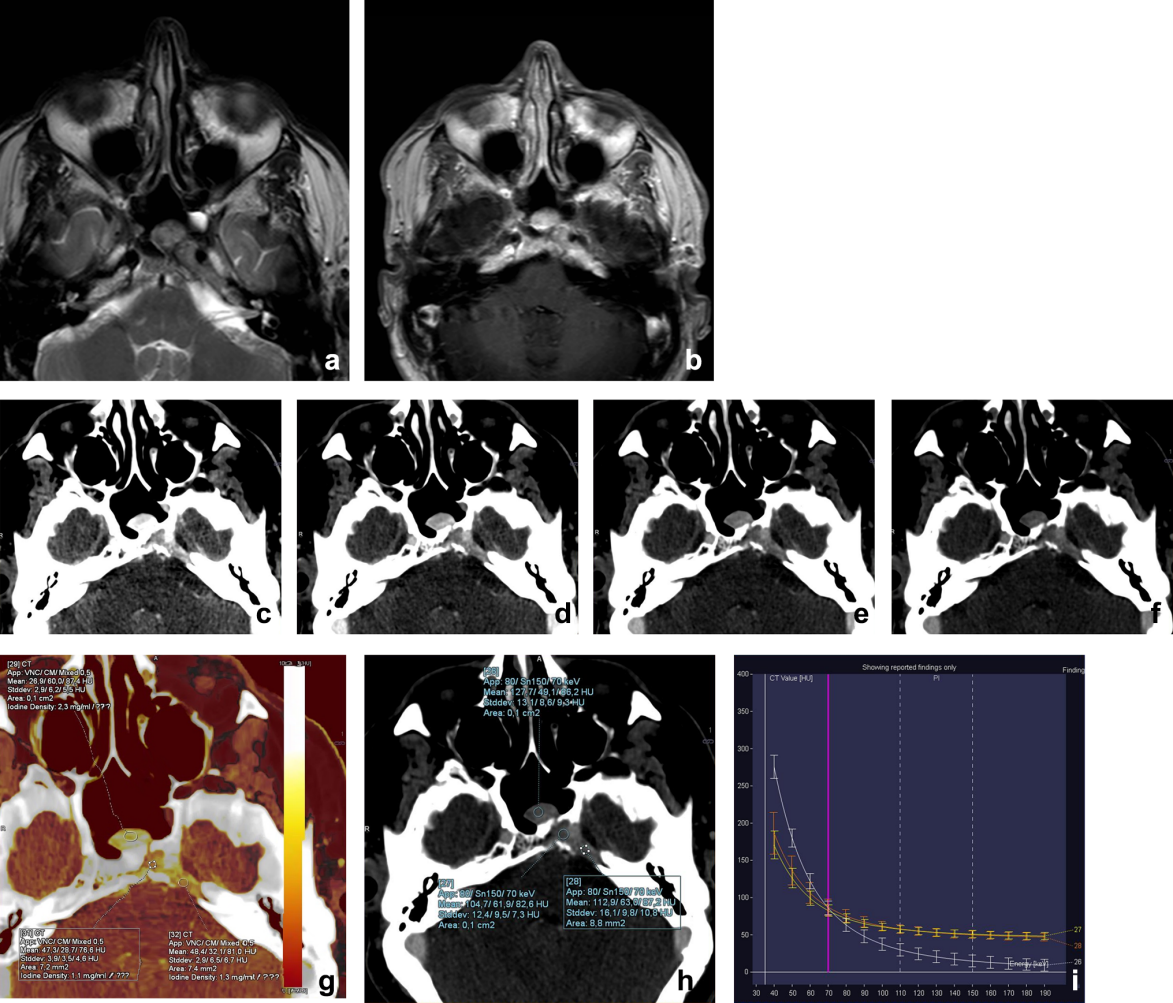
A 48-year-old male presented with a traumatic skull base lesion. Initial CT demonstrated a lesion at the petroclival fissure, apex, and sphenoid sinus. MR with T2-weighted (**a**) and T1-weighted post-gadolinium images (**b**) showed the presence of the lesion with relatively low T2-signal and enhancement after gadolinium. By performing a transnasal biopsy of the sphenoid part, it would be feasible to obtain a histopathological diagnosis of the skull base lesion. On CT, the lesion is well appreciated with osteolysis of the petrous apex and enhanced after iodinated contrast. However, after mono-energetic reconstructions (**c**–**f**, 40–80 keV) and iodine fusion imaging, the lesion consists of two parts, with the medial part being more enhancing at lower keV, with higher iodine uptake at fusion imaging (**g**, **h**). The graphs of the spectral curves (**i**) demonstrate two different attenuation curves. It was concluded that the medial part of the lesion was different from the lateral part and probably herniated pituitary after trauma. The more lateral lesion of the skull base still has no definitive diagnosis, because of the difficulty in accessing for biopsy. Thus far, no growth of the lesion is noted during follow-ups

Currently CT and MRI are the standard image modalities used for primary staging of head and neck squamous cell carcinoma (HNSCC). Accurate diagnostic staging is essential for proper treatment of HNSCC and patient survival. Important factors affecting initial treatment of HNSCC include primary site, size, location, proximity to bone or cartilage, status of cervical lymph nodes, previous treatment, and histology [38]. Both CT and MRI report acceptable sensitivity and specificity for HNSCC staging in literature [39, 40]. The evaluation of the tumor and infiltration of surrounded anatomical structures can be challenging. DECT, with its advantages in material characterization and differentiation, can be beneficial in primary HNSCC imaging. With the presence of two datasets at different kVp settings a variety of reconstructions can be made (Fig. 7).

Tawfik et al. demonstrated that images with a weighted average (WA) ratio of 0.6 and 0.8 in which the percentage of the lower energy is relatively high, the delineation of head and neck tumors significantly improved compared to the WA 0.3 images which resemble a SECT of 120 kVp. Also the subjective image quality of the WA 0.6 images was superior to the WA 0.3 images [41]. In a more recent study, Scholtz et al. found the same trend of better tumor enhancement with increasing WA of 0.3, 0.6 and 0.8 in linear-blended imaging. In addition, Scholtz et al. compared a nonlinear image blending (“optimum contrast” application) to the different linearly blended images. In nonlinear-blended images the advantages of lower SNR at higher energies and the increased CNR in the lower energy spectrum are combined. This resulted in increased lesion conspicuity, while maintaining a good SNR [15, 42]. Furthermore, subjective overall image quality favored nonlinear-blended images [16].

In another study of Scholtz et al., blended 120-kVp images were compared with 80-kVp acquisition images. The mean tumor enhancement was higher at the 80-kVp acquisition. Both were rated as good subjective image quality, but blended 120-kVp images were rated superior to those of 80 kVp. The presence of image noise was increased at the 80-kVp images, with a higher presence of metallic artifacts [43].

In a prospective study of Toepker et al. the identification of tumor margins in patients with oral cancer was compared in 80-, 140-kVp, mixed, and ‘optimum contrast’ (OC) DECT images. Low-kVp, mixed, and OC images all received good-to-excellent scores in image quality, while 140-kVp images were rated as moderate to low. Contrast at the tumor margins was the highest for 80-kVp, mixed, and OC images compared with 140-kVp images but the low-energy images showed the highest image noise and were more prone to metal artifacts than the mixed and OC images. SNR was more favorable in mixed images [44].

**Fig. 8:**
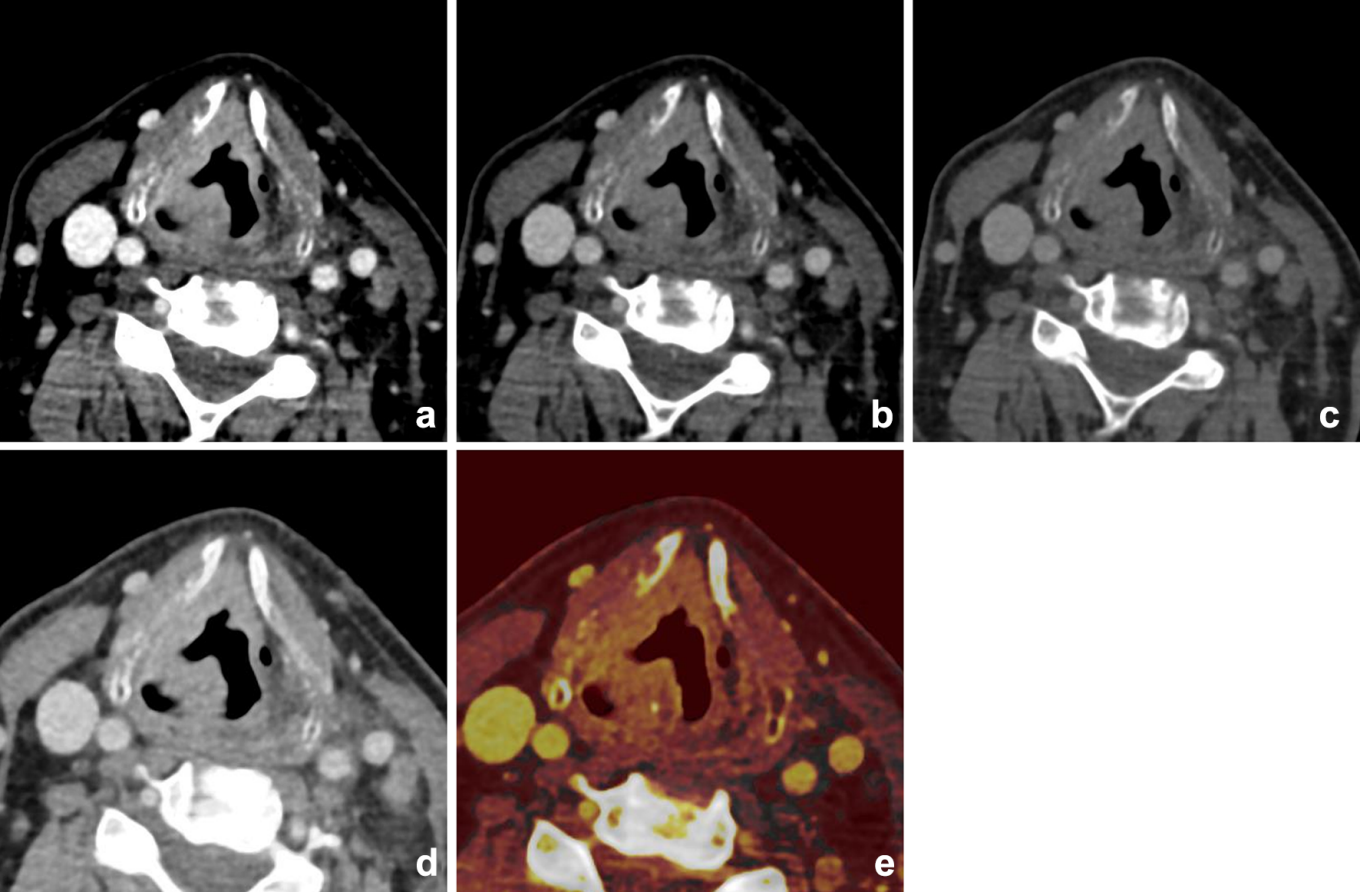
Supralaryngeal carcinoma of the right hemilarynx in a 74-year-old male. Increased conspicuity of the tumor was shown at the lower virtual mono-energetic reconstructions (40 (**a**), 55 (**b**), and 70 (**c**) keV). Note the increased differences between tumor and strap muscles at lower-keV settings, compared with mixed imaging (**d**) and higher-keV settings. These differences were even more enhanced on iodine fusion imaging (**e**). Also the extra-laryngeal extension was more easily appreciated at the iodine fusion images

**Fig. 9:**
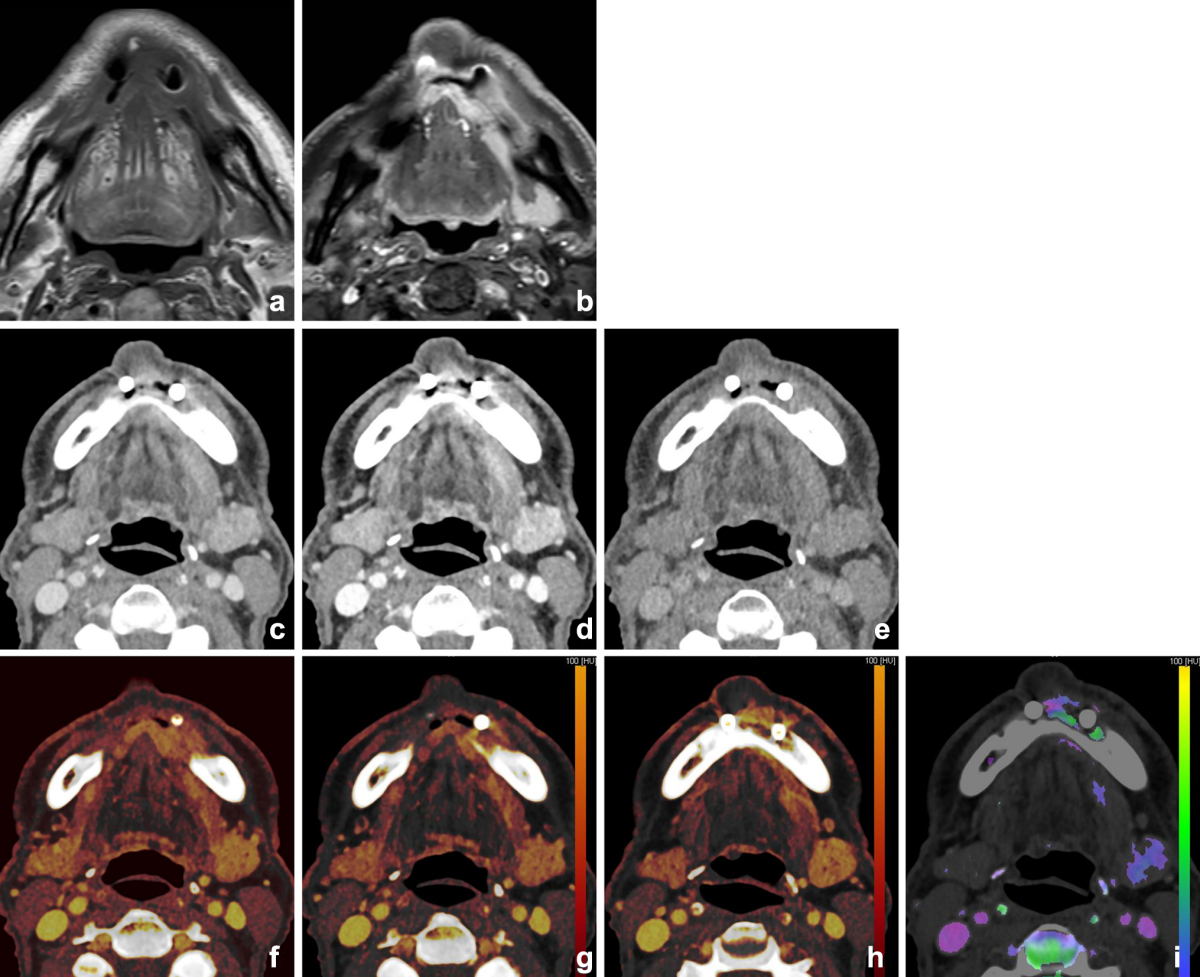
A male with previous laryngeal cancer and oral implants presented with suspicion of oral cavity SCC at the site of the implants. MR showed considerable distortion and interpretation difficulties at the tumor site (T1-weighted (T1-w) (**a**). Fat-supressed T1-w after gadolinium (**b**) demonstrated abnormal signal and enhancement; however, delineation was challenging due to artifacts. 5 mm DECT with soft kernel at 50% linear blending (**c**), 90% (**d**), and 10% (**e**) blending demonstrated the implants and osseous destruction of the mandible, better visualized at higher energies. Tumor enhancement was, however, difficult at mixed imaging, but was easily visualized at iodine fusion imaging (**f**–**h**). Bone marrow edema is demonstrated at BME imaging (**i**)

VMI can also be applied for better tumor delineation in head and neck oncology [18] [45••]. Because of higher attenuation of iodine in lower-keV VMI, superior tumor contrast by iodine uptake can be achieved; this, however, can occur at the cost of higher image noise (Figs. 8, 9). Wichmann et al. investigated the value and image quality of VMI at different energy levels in patients with HNSCC. Objective enhancement of SCC lesion peaked in 40-keV, followed by 60-keV reconstructions, but 60-keV VMIs were subjectively more favorable in image quality and tumor delineation. Compared with linearly blended images, with a WA 0.3, VMI of 60 keV seemed to be superior in tumor attenuation and CNR [18].

Albrecht et al. evaluated the advanced application of VMI, (Mono+) and compared the images to linear-blended WA 0.3 DECT images in patients with head and neck cancer. The highest tumor attenuation was found at 40 keV with a superior CNR compared with the WA 0.3 images. However, subjectively VMI at 55 keV was preferred regarding image quality and tumor delineation [21]. Lam et al. evaluated VMI reconstructions of the head and neck with a single source fast-kV switching DECT. They found the highest SNR at 65-keV VMI for all tissues in head and neck imaging, but better tumor delineation and CNR at 40-keV VMI [45••]. Based on their findings, Lam et al. recommend the use of a multiparametric approach with 65-keV VMI for general assessment of the neck, supplemented by 40-keV VMI for better tumor differentiation to the surrounding soft tissue of the head and neck. Optimal SNR can thus vary depending on the scanner type and postprocessing techniques applied.

### Bone Invasion

#### Invasion of Thyroid Cartilage

Accurate detection of cartilage invasion is of great importance for the appropriate treatment choice of hypopharyngeal and laryngeal squamous cell carcinoma (SCC). Tumors without or with limited cartilage invasion can be treated with organ-preserved interventions including CO_2_-laser, minimally invasive surgery or (chemo)radiotherapy. Tumors with evident cartilage invasion require more aggressive treatment, frequently resulting in total laryngectomy, which may significantly impair the patient’s quality of life [46].

Both CT and MRI imaging are currently used for the evaluation of cartilage invasion, each having its advantages and limitations. At CT, sensitivity and specificity of detection of cartilage invasion depend on the various diagnostic criteria of sclerosis, erosion, lysis, and extra-laryngeal spread [47, 48]. Becker et al. reported that sclerosis was the most sensitive criteria in all cartilages, but it could be due to reactive inflammation. An optimal combination of criteria yielded an overall sensitivity of 91%, with a specificity of 68% [47, 48]. One of the problems with the use of SECT-imaging is the resemblance of attenuation of HNSCC with the attenuation of non-ossified laryngeal cartilage, making it difficult to accurately distinguish subtle cartilage invasion.

In a few recent studies, DECT has been shown to be beneficial in the evaluation of cartilage invasion by laryngeal and hypopharyngeal squamous cell carcinoma (SCC). Kuno et al. evaluated the combination of iodine overlay maps (IOM) and 0.3 WA images to evaluate cartilage invasion in hypopharyngeal and laryngeal SCC. IOM combined with WA imaging significantly improved the specificity of detection of laryngeal cartilage invasion compared with WA imaging alone with, respectively, 96 versus 70%, while sensitivity remained at 86%. Furthermore, the inter-observer reproducibility of evaluating cartilage invasion also improved [49].

**Fig. 10:**
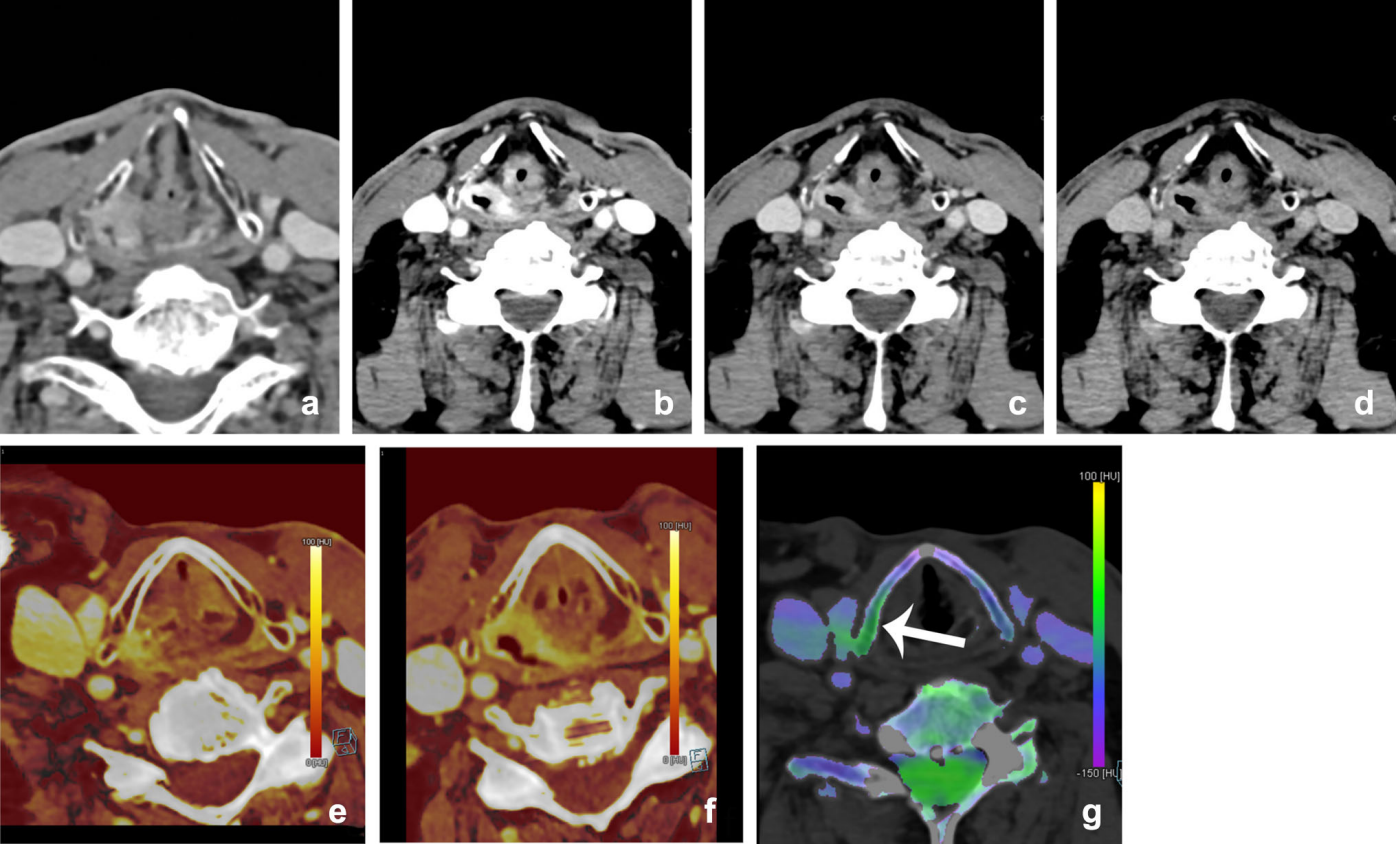
Different reconstructions from one dataset. DECT of a 77-year-old male showed a right-sided piriform sinus SCC (**a**, mixed), suggestive of thyroid invasion. The tumor delineation is better depicted at the lower-energy images of the VMI (**b**: 40 keV; **c**: 70 keV; and **d**: 100 keV), and on the iodine fusion images (**e**, **f**). Bone marrow edema (BME) image (**d**), which demonstrates edema at the thyroid cartilage. Visualization of direct tumor invasion is the easiest way to demonstrate cartilage invasion; this can be demonstrated made more easily by means of lower-energy images from VMI and iodine fusion images. Demonstration of edema of the thyroid in virtual non-calcium images can serve as an additional argument for invasion of the thyroid (*arrow*)

The use of VMI in the assessment of cartilage invasion has been recently evaluated by a retrospective study of Forghani et al. Thirty patients with laryngeal or hypopharyngeal SCC and 10 healthy patients underwent fast-kV switching DECT. It was shown that tumorous cartilage has a significantly different spectral HU curve than normal non-ossified thyroid cartilage on VMIs equal to or higher than 95 keV [50, 51]. Based on these studies, an improvement in accurate staging with DECT in the future seems feasible (Fig. 10).

### Bone Marrow Edema

In general, bone marrow edema (BME) is best visualized by MR imaging techniques. With the DECT virtual non-calcium (VNCa) technique, it is possible to assess bone marrow edema with CT.

In the head and neck area, the presence of dental restorations, irregular tooth sockets, periapical and periodontal infection, other inflammatory reactions, and edema or sclerosis can give false positive results on both CT and MR imaging [52–54]. In daily practice, a combination of CT and MR imaging is often used to visualize subtle bone involvement in oncology, osteomyelitis, and osteonecrosis. No human studies in the head and neck area concerning DECT BME have been published.

**Fig. 11:**
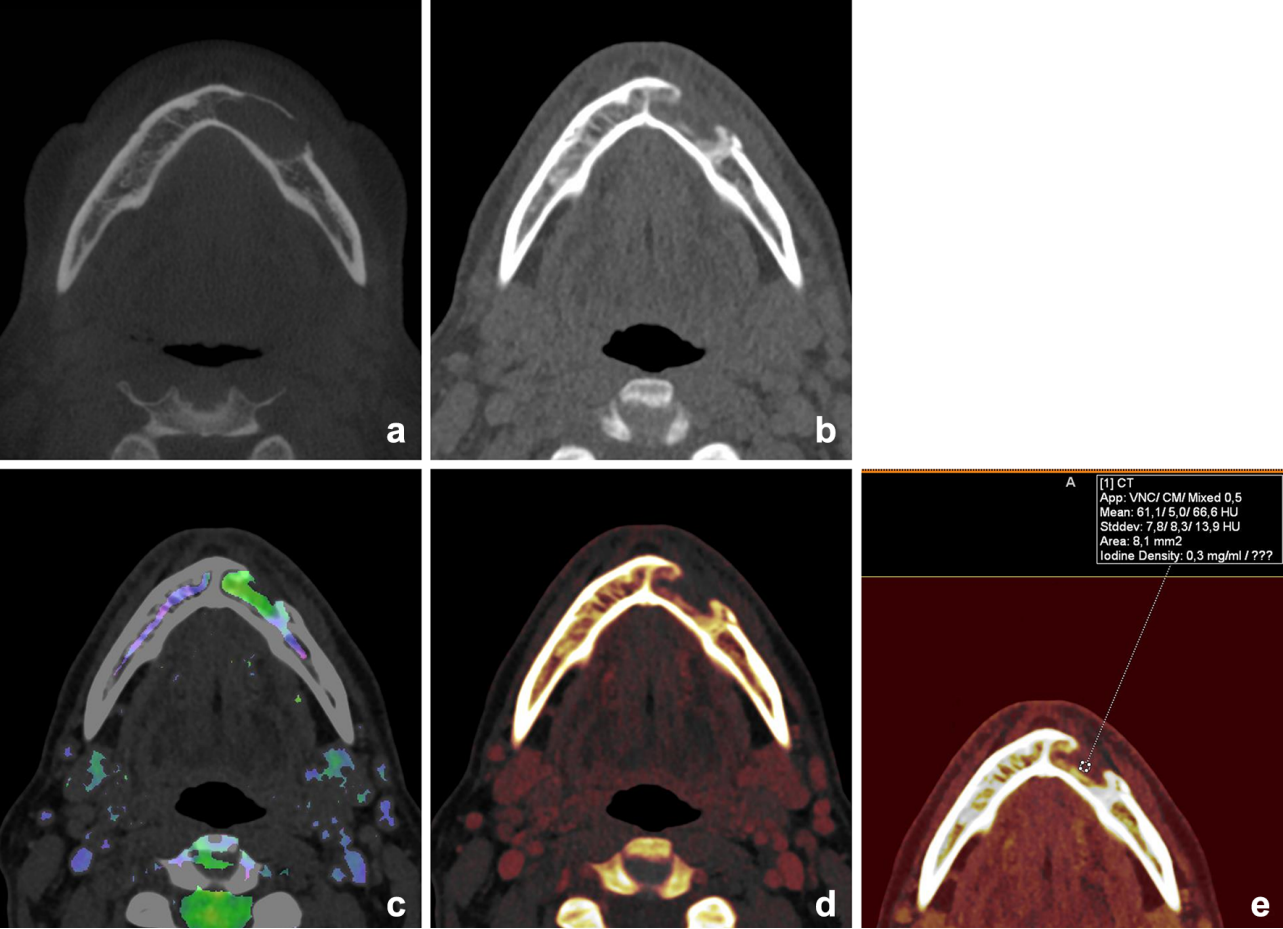
A 50-year-old female underwent cystostomy for a left-sided keratocyst of the mandible (**a**). After 6 months, therapy was evaluated by DECT(**b**, mixed). A residual lesion was found, with some residual edema (**c**, BME) and reparative bone apposition, but without signs of enhancement (**d**, **e**, iodine fusion (iodine concentration 0.3 mg/ml))

**Fig. 12:**
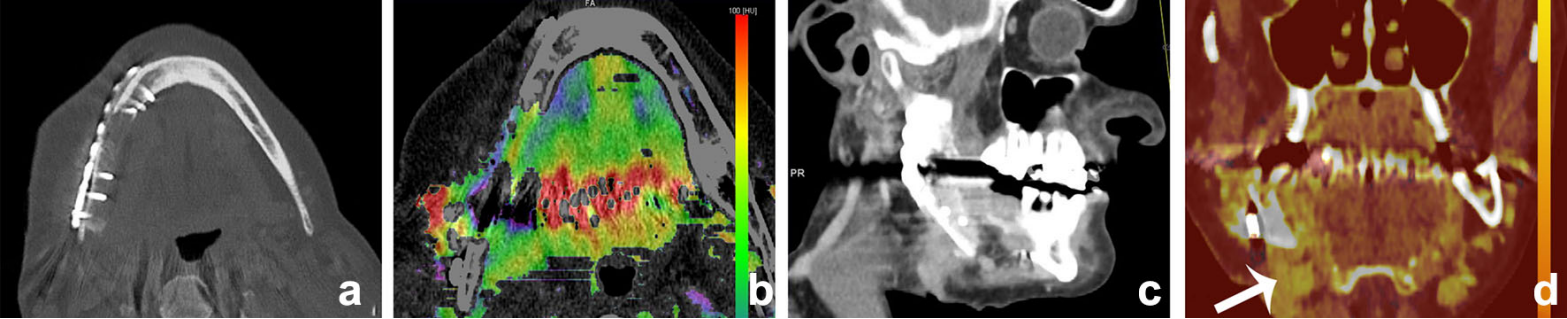
A 60-year-old male presented with a persistent fistula after mandibular reconstruction, due to chronic osteomyelitis of the mandible. Depicted are 5 mm Maximum intensity projections of the mandible, showing metallic hardware next to an osteolytic mandible (**a**). BME images demonstrate bone marrow edema at the right mandible (**b**). The fistula is demonstrated at (**c**) and clearly enhanced at iodine fusion images at (**d**, *arrow*)

**Fig. 13:**
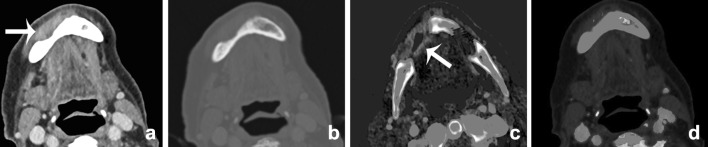
A 90-year-old male was referred for staging of a gingival tumor originating at the right mandible. An MRI was contraindicated, and a DECT of the oral cavity was performed. Mixed images in soft tissue kernel (**a**) and bone kernel (**b**); iodine fusion images (**c**), and BME reconstructions (**d**). An enhancing tumor was found at the right mandible (**a**, *arrow*) overlying an area of eroded or remodeled mandible (**b**) with sclerosis. Iodine fusion images additionally suggested invasion of the floor of the mouth, which was not seen on the mixed images (**c**, *arrow*). BME images did not show bone marrow edema. At histopathology, no osseous invasion was found

Poort et al. investigated DECT as imaging technique for BME in osteoradionecrosis of the mandible in Göttingen mini-pigs. In this study, DECT was found to be an adequate single-modality imaging technique for simultaneous detection of structural bone changes, such as cortical disruptions, and BME. Further research is needed to investigate if DECT BME in the head and neck region is reliable [55]. Preliminary findings at our department demonstrate that it is feasible to show bone marrow edema in the human mandible (Figs. 9, 10, 11, 12 and 13).

### Cervical lymph Node Imaging

The presence of metastatic lymph nodes in the neck significantly reduces the 5-year disease-specific survival rate in patients with HNSCC [56]. Since clinical physical examination of the neck has limitations, imaging is important, to confirm the N0 status of the neck; to document lymphadenopathy contralateral to clinically palpable disease; and to assess the regional extent of disease, especially in relation to neurovascular structures and nodal surveillance for follow-up [57, 58].

**Fig. 14:**
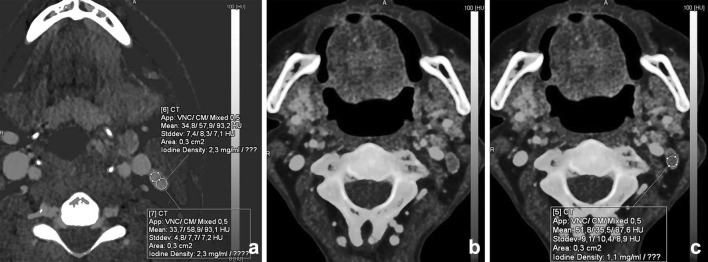
Differentiation between malignant and benign or inflammatory lymph nodes is feasible with DECT. A patient with a parapharyngeal abscess demonstrated a level-2 lymph node; iodine uptake was 2.3 mg/ml (**a**); this in contrast to the iodine concentration of a lymph node in a patient with oropharyngeal squamous cell carcinoma of the vallecula (iodine concentration 1.1 mg/ml). Malignant lymph nodes have lower iodine uptake than normal or inflammatory lymph nodes, as is demonstrated in (**b**) and (**c**)

Several studies showed that DECT can be useful in lymph node imaging of the neck with good image quality and dose reduction [11, 23, 59–62]. Besides good image quality and lower image noise, functional and metabolic parameters can be extracted from DECT datasets, as, e.g., iodine quantification (Fig. 14). The iodine uptake in lymphatic tissue may be utilized as a surrogate marker for perfusion (hypoxia) and angiogenesis [63, 64]. Liang et al. showed that ratios of the slope of the spectral curves between the lesion and the lymph nodes were significantly different in metastatic lymph nodes compared with non-metastatic lymph nodes [58]. Tawfik et al. observed that iodine parameters were significantly lower in metastatic lymph nodes than those in normal or inflammatory lymph nodes. The iodine content (mg/ml), directly quantifying the amount of iodine in each voxel, seemed more useful than the iodine overlay (HU). Using iodine content to differentiate metastatic nodes from normal and inflammatory nodes, a threshold value of 2.85 mg/ml yielded a sensitivity of 85% and a specificity of 87.5% [65•].

### Differentiating Recurrent Disease from Normal Posttreatment Changes

**Fig. 15:**
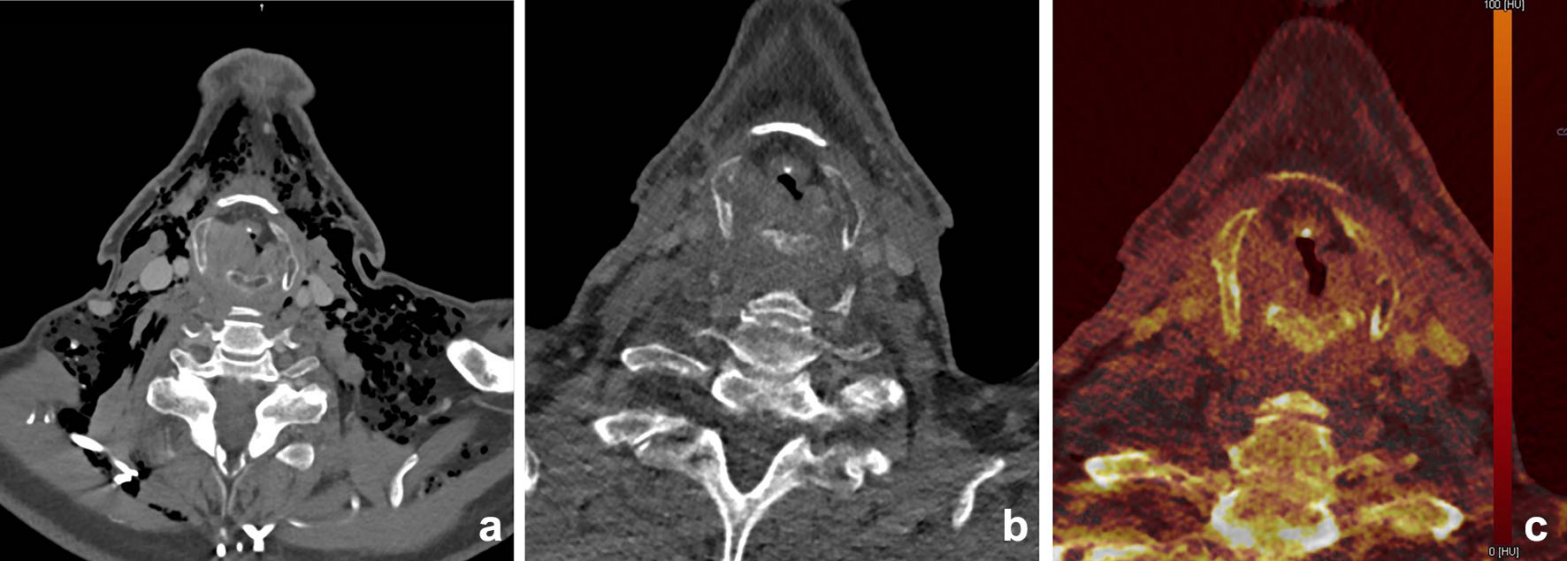
An 81-year old male patient with a hypopharyngeal carcinoma from the piriform sinus, presented 6 months after chemoradiation to evaluate therapy response. The initial CT with the presence of a bilateral tumor of the piriform sinus and massive (postoperative) subcutaneous emphysema (**a**). A mixed DECT at one year (**b**) was made for evaluation of therapy effect and exclusion of residual tumor. Bilateral swelling is present, without clear enhancement at mixed imaging. At the iodine overlay images (**c**), no increased iodine uptake was present; benign posttreatment changes without residual or recurrent tumor were concluded

Only a limited number of studies have been focusing on DECTs ability to differentiate between benign and malignant changes in patients with a history of neck malignancy. In a study of Srinivasan et al., the spectral HU curve was shown to be promising for differentiating benign posttreatment changes from malignant neck pathologies [66] (Fig. 15). More recently, Yamauchi et al. had similar results in their study for the spectral HU curves and corresponding results for the iodine concentration [67•]. In addition to the earlier study by Srinivasan, the latter authors compared the spectral HU curves at 40 and 70 keV: the first theoretically showing the highest iodine concentration and the higher-energy-level curve representing a standard MDCT with a 120-kVp polychromatic X-ray beam. Comparison of the two showed better results for the curve at 40 keV.

### Infection and Inflammation

**Fig. 16:**
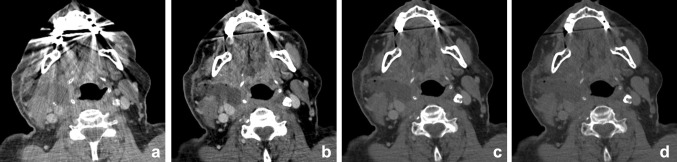
Infection/abscesses: increased conspicuity at lower keV. A 65-year-old-male patient presented with a neck mass and a c-reactive protein of 180 μg/ml one week after carotid endarteriectomy. Mixed/blended imaging showed right-sided swelling of the neck (**a**). Anterior of the sternocleidomastoid muscle, a fluid collection was visible, without obvious rim enhancement at mixed imaging. **b**–**d** The virtual mono-energetic reconstructions of 40 (**b**), 55 (**c**), and 70 (**d**) keV. At higher keV, SNR was increased, but showed decrease of lesion conspicuity. At 40 keV, the lesion conspicuity and rim enhancement of the lesion were most optimal and suggestive of abscess formation. Small *air bubbles* were visible at the anterior part of the lesion

Peritonsillar inflammation and abscess may present at any age, but they have the highest incidence rates in the adolescent population [68]. Therefore, especially in this patient group, scanning should be performed according to the ALARA principle. Scholtz et al. described the benefits of low-voltage images in peritonsillar abscess (PTA) compared with linearly blended 120-kVp images [16]. First of all, they demonstrated an improved delineation of the PTA in 80-kVp images compared with the 120-kVp images. They reported a significant increase in SNR and rim-to-abscess CNR (Fig. 16). Subjectively, the image sharpness was significantly better in the 80-kVp images. Another possible advantage is the increased attenuation in adjacent vessels. This might improve operation planning in order to prevent bleeding in case of incision and drainage of the PTA. However, further studies must evaluate whether 80-kVp scanning alone is sufficient in detection of PTA for additional dose reduction. Other possible advantages of DECT will still require the high-voltage scan.

**Fig. 17:**
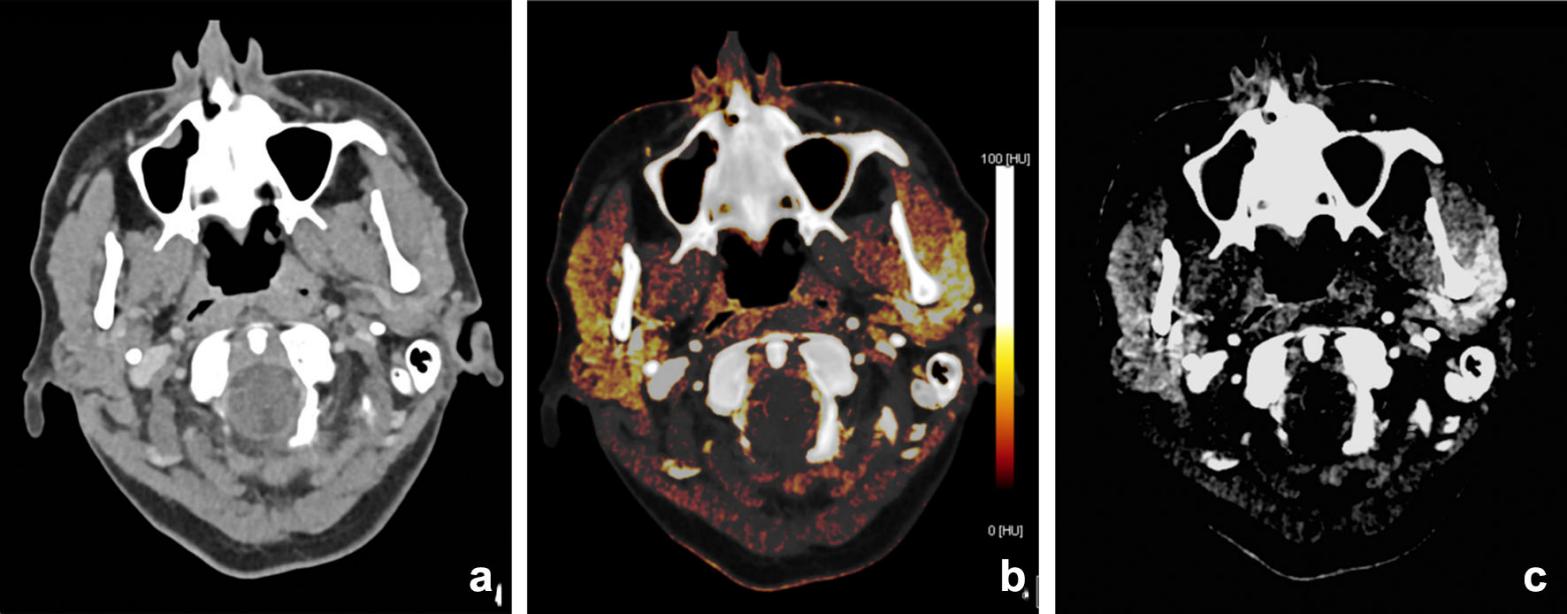
A 48-year-old female with poor dental status presented with facial swelling and painful mouth opening. DECT was performed to rule out dental or neck abscesses, or arthritis of the temporomandibular joint. Mixed, 120-kVp-like images (**a**) demonstrated, besides a slight asymmetry no obvious abnormalities or fat stranding. The scan was initially interpreted as normal. However, Iodine map (**b**) and iodine fusion (**c**) images demonstrated clearly increased uptake of iodine at the superficial part of the parotid gland. Parotitis of the superficial lobe of the parotid gland was concluded

**Fig. 18:**
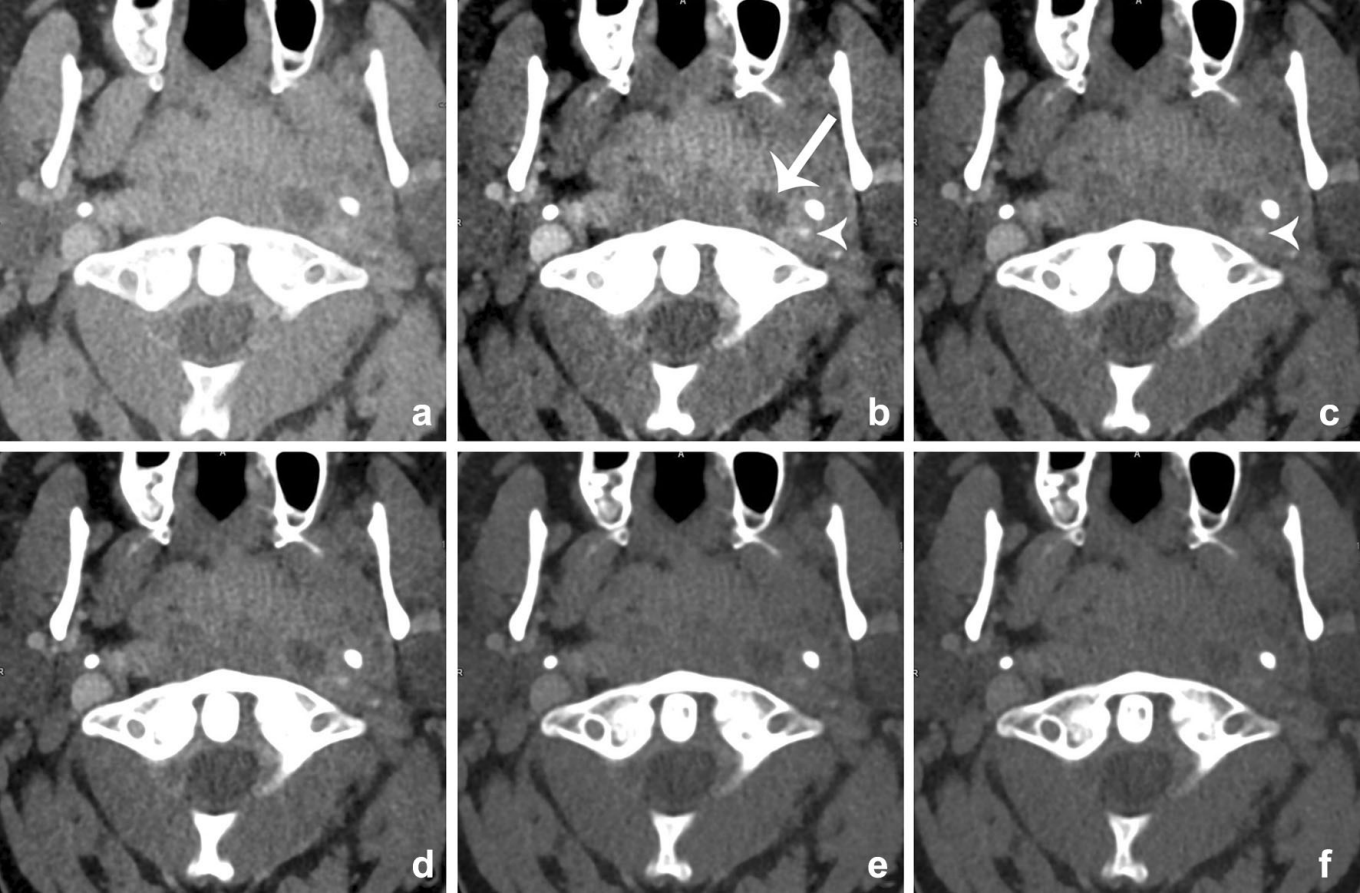
Images of a young male patient with prevertebral abscesses. Blended images (50%) (**a**) and VMIs (**b**–**f**). VMIs demonstrate improved visualization of the infiltration and abscesses at lower energies (*arrow*): 40 keV (**b**), 50 keV (**c**), 60 keV (**d**), 70 keV (**e**), and 80 keV (**f**). There is better visualization of the internal carotid artery at lower mono-energetic reconstructions, compared with blended image and higher-energy images, although at the cost of increased noise (*arrowhead*)

Wichmann et al. reported positive results in improved detection of sialoadenitis in three patients [60]. DECT of inflammatory diseases of the head and neck are thus far scarcely evaluated. Nevertheless, the results suggest a promising contribution of DECT due to the increased delineation, which can aid in an early detection and delineation of inflammation and abscesses (Figs. 17, 18).

### Parathyroid Tumors: Parathyroid Adenomas

In patients with primary parahyperthyreoidism, adenomas are the most common cause. They are usually juxta thyroid single lesion, but multiple lesions and/or ectopic locations can occur. Treatment is surgical and has changed from bilateral exploratory to minimal invasive surgery. In order to be successful, preoperative identification and localization of the parathyroid adenomas is essential. A combination of sestamibi scanning and ultrasonography has proven effective with the addition of a third technique if results are not concordant [69].

**Fig. 19:**
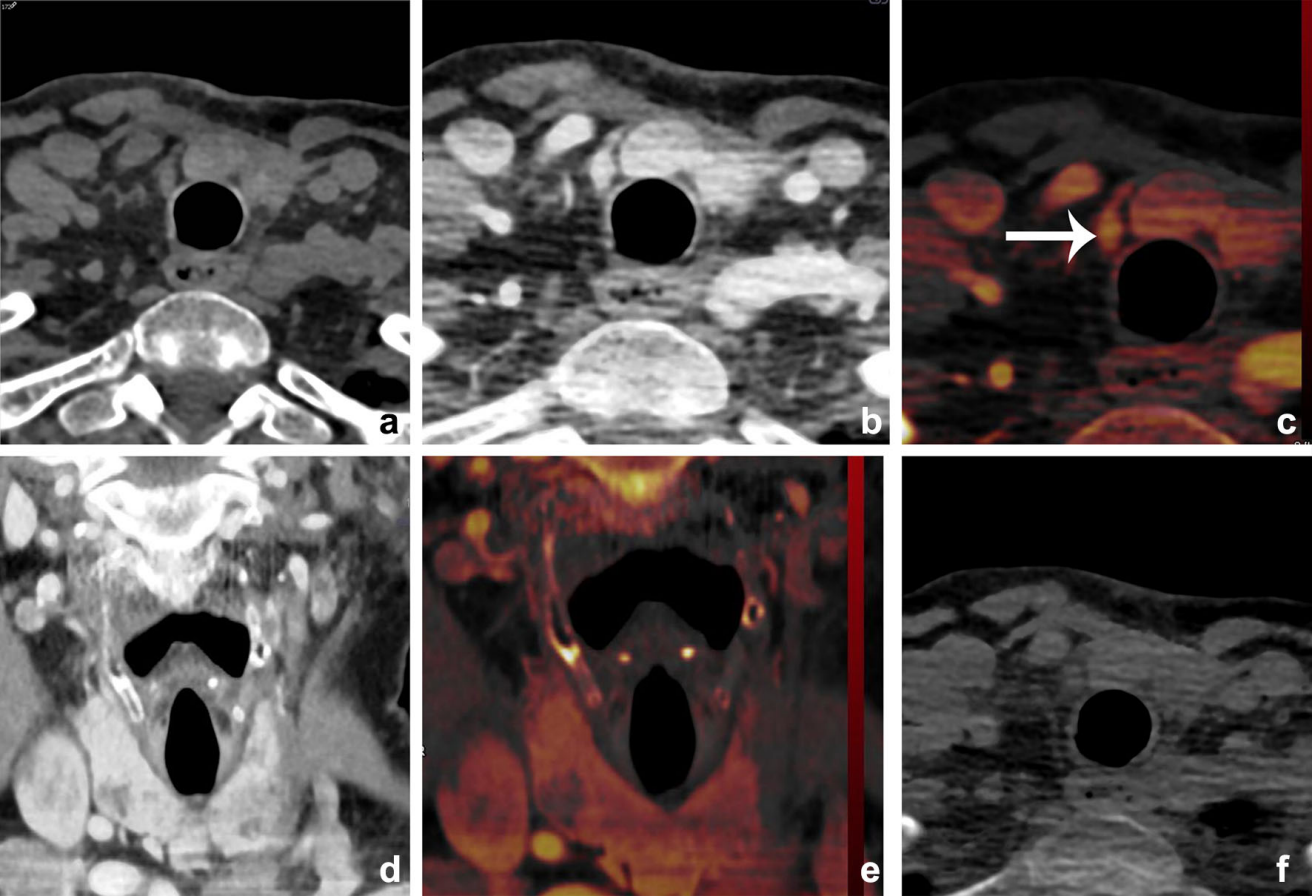
A 63-year-old female presented with primary hyperparathyroidism. Initial Choline PET imaging and MIBI scanning were negative. A multiphase CT showed a small enhancing lesion just caudal to the right thyroid lobe, which was proven a parathyroid adenoma (*arrow*). Axial noncontrast images (**a**), 30-sec axial-mixed images (**b**), iodine fusion images (**c**), and coronal mixed (**d**), and fusion (**e**) images. A Virtual noncontrast image is shown at (**f**), the latter demonstrating more noise and streak artifacts than the true nonenhanced scan, although still diagnostic. Noncontrast CT: 80 kVp 277 mAs; CTDI 5.34 mGy; DLP 113.4 mGycm. DECT 30 s: 80/150 kVP 61/31 mAs; CTDI 5.52 mGy; DLP 113.0 mGycm

Multiphase CT is accurate in localization of parathyroid adenomas due to different perfusion characteristics of thyroid gland, lymph nodes, and parathyroid adenomas. Due to awareness of radiation dose and diversity in protocol, the use was limited [70]. Gafton et al. showed that parathyroid hormone-secreting lesions can be differentiated from other soft tissue structures by evaluating attenuation characteristics in arterial and venous phase. They could reduce radiation exposure by limiting the protocol to 2 phase imaging [71]. However, some believe this limitation in protocol reduces diagnostic confidence due to lack of a complete multiphase images. DECT is recommended to provide a more complete multiphase examination using VNC images and still reduce radiation exposure [72]. Forghani et al. demonstrated statistically significant differences in several DECT parameters partly depending on scan phase [73]. The arterial phase showed greater contrast between adenomas and lymph nodes, whereas the 55-s phase showed greater contrast between adenomas and thyroid gland. Although evidence is still limited, results indicate that multiphase DECT evaluation of parathyroid adenomas can enhance diagnostic accuracy. Moreover, the ability of DECT to calculate VNC images could theoretically lower radiation dose (Fig. 19).

### Thyroid Disease

Management of thyroid nodules remains challenging. DECT has been used to discriminate between benign and pathological nodules in a few studies by Li et al. [74–76]. These studies showed a difference in iodine uptake between benign and pathological nodules. Intralesional hemorrhage could be differentiated from solid nodules. Moreover, they could discriminate normal and metastatic lymph nodes from papillary thyroid carcinoma. It remains a matter of debate whether it is wise to use CE-CT in ruling out thyroid carcinoma.

### Radiotherapy Planning

In radiotherapy, CT plays an important role for dose calculation in treatment planning, because of its relatively easy calibration of HU to electron densities [77, 78]. The possibility to calculate Zeff and electron density was already described by Hounsfield at the initial descriptions of DECT in 1973. It was demonstrated that a clinical DECT scanner was able to extract Zeff, and density *ρ* of different tissue substitutes, next to ΔHU and *ρe* [79–81]. This suggested that when a large quantity of high-density and high atomic number structures are in the planning field, DECT-derived calculations show accurate and reliable inhomogeneity corrections in RT treatment planning [82].

There is an increasing interest in proton therapy because of its higher dose conformity and sparing of organs at risk compared with intensity-modulated radiation therapy [83]. Hudobivnik compared the proton therapy treatment planning of head tumors at the skull base to calculate the stopping powers while using SECT and DECT [84]. They confirmed a higher accuracy for DECT in their surrogate patients using a pencil beam algorithm. Zhu et al. confirmed in a phantom the dosimetric advantages in proton therapy treatment planning with DECT over the current approach based on SECT [85]. Whether this is clinically relevant needs to be investigated in future.

## Conclusion

The use of DECT in head and neck imaging has been growing in the recent years. The advantages of additional DECT reconstructions at a comparable radiation dose are recognized by an increasing number of head and neck specialists. VMI and iodine characterization of DECT may play a major role in patients with HNSCC in detection and delineation of the tumor, resulting in more accurate staging. It can differentiate between malignant and benign lymph nodes based on iodine concentration, as well as between benign posttreatment changes and recurrent disease. VMI at higher keV is useful for reduction of metallic artifacts. Three material differentiation algorithms for identification of iodine and calcium can be used to assess cartilage and bone marrow infiltration, the latter being a new application in head and neck DECT. Imaging of infection and inflammation can be mitigated with DECT, and differential diagnosis can be facilitated with the use of spectral curves.

With the use of DECT, the inherent image information is more obvious due to the application of material characterization and differentiation, while maintaining a lower or equal radiation dose. This can especially be helpful in a difficult anatomical area like the head and neck.

## Abbreviations

ALARA: As low as reasonably achievable
BME: Bone marrow edema
CNR: Contrast-to-noise ratio
CT: Computed tomography
CTA: Computed tomography angiography
CE-CT: Contrast-enhanced CT
DECT: Dual-energy CT
HNSCC: Head and neck squamous cell carcinoma
HU: Hounsfield units
IMAR: Iterative metal artifact reduction
IOM: Iodine overlay map
keV: Kilo electron volt
kVp: Peak kilo electron volt
MDCT: Multidetector CT
MR(I): Magnetic resonance imaging
OC image: Optimal contrast image
OPTkeV: Optimized image quality keV
PET: Positron emission tomography
PTA: Peritonsillar abscess
SECT: Single energy CT
SNR: Signal-to-noise ratio
SCC: Squamous cell carcinoma
VMI: Virtual mono-energetic imaging
VNCa: Virtual non-calcium
VNC: Virtual noncontrast
WA: Weighted average
